# Controlled expression of functional miR-122 with a ligand inducible expression system

**DOI:** 10.1186/1472-6750-10-76

**Published:** 2010-10-20

**Authors:** Cathy M Shea, George Tzertzinis

**Affiliations:** 1 RNA Biology Division, New England Biolabs, 240 County Road, Ipswich, MA, USA

## Abstract

**Background:**

To study the biological function of miRNAs, and to achieve sustained or conditional gene silencing with siRNAs, systems that allow controlled expression of these small RNAs are desirable. Methods for cell delivery of siRNAs include transient transfection of synthetic siRNAs and expression of siRNAs in the form of short hairpins using constitutive RNA polymerase III promoters. Systems employing constitutive RNA polymerase II promoters have been used to express miRNAs. However, for many experimental systems these methods do not offer sufficient control over expression.

**Results:**

We present an inducible mammalian expression system that allows for the conditional expression of short hairpin RNAs that are processed *in vivo *to generate miRNAs or siRNAs. Using modified nuclear receptors in a two hybrid format and a synthetic ligand, the Rheoswitch system allows rapid and reversible induction of mRNA expression. We evaluated the system's properties using miR-122 as a model miRNA. A short hairpin encoding miR-122 cloned into the expression vector was correctly processed to yield mature miRNA upon induction with ligand and the amount of miRNA produced was commensurate with the concentration of ligand. miR-122 produced in this way was capable of silencing both endogenous target genes and appropriately designed reporter genes. Stable cell lines were obtained, resulting in heritable, consistent and reversible expression of miR-122, a significant advantage over transient transfection. Based on these results, obtained with a microRNA we adapted the method to produce a desired siRNA by designing short hairpins that can be accurately and efficiently processed.

**Conclusion:**

We established an Inducible expression system with a miR-122 backbone that can be used for functional studies of miRNAs and their targets, in heterologous cells that do not normally express the miRNA. Additionally we demonstrate the feasibility of using the miR-122 backbone to express shRNA with a desired siRNA guide strand for inducible RNAi silencing.

## Background

There is a growing awareness of the significance of small RNAs in biology, which has led to increased use of small RNAs as tools in biological research. For example, microRNAs (miRNAs) are small non-coding RNAs that regulate gene expression by reducing the stability or the translation of partially complementary mRNA [1,2] and up to 30% of human genes may be regulated by miRNAs [3,4]. RNAi, mediated by short double-stranded RNAs (siRNAs), has become a powerful tool for analyzing gene function through targeted gene knock down [5]. Improved methods for controlled expression of small RNAs in the cell will advance the study of their roles in biological processes.

miRNA genes are transcribed by RNA polymerase II (pol II) and the primary transcript is processed *in vivo *to yield first a short hairpin, and finally a 21-23 nt miRNA [6]. Synthetic siRNA can be synthesized *in vitro *and delivered to cells by transfection. Alternatively, short RNA hairpins that mimic a miRNA precursor can be expressed in the cell using either plasmid or viral vectors. The resulting transcript is processed *in vivo *to yield a small RNA that can function as an siRNA, or a miRNA, inducing specific degradation of targets, similar to transfected siRNA [7,8]. For certain RNAi applications, expression of short hairpins offers advantages over transient transfection of siRNA. Expression vectors can be transiently transfected or integrated into the cellular genome to create stable cell lines. The latter method provides consistent, long-term expression of the short hairpin as compared with transient transfection of siRNA. Early expression vectors used the U6 and H1 RNA polymerase III (pol III) promoters which use discrete initiation and termination marks [9,10]. Constitutive pol II promoters, such as CMV [6,7] or UbC [8], have also been used (reviewed in [9]). But in many cases, such as analysis of essential genes, a conditional expression system that can produce siRNA or miRNA on demand or for a limited time is required. While pol III promoters allow a high level of expression they are naturally constitutive. Efforts to engineer pol III systems under drug-mediated control (e.g. Tet-based systems) have compromised either the tight repression of expression in the OFF state [11] or the high level of expression in the ON state [12]. The advantage of the Tet system is that expression is reversible upon drug withdrawal.

**Figure 1:**
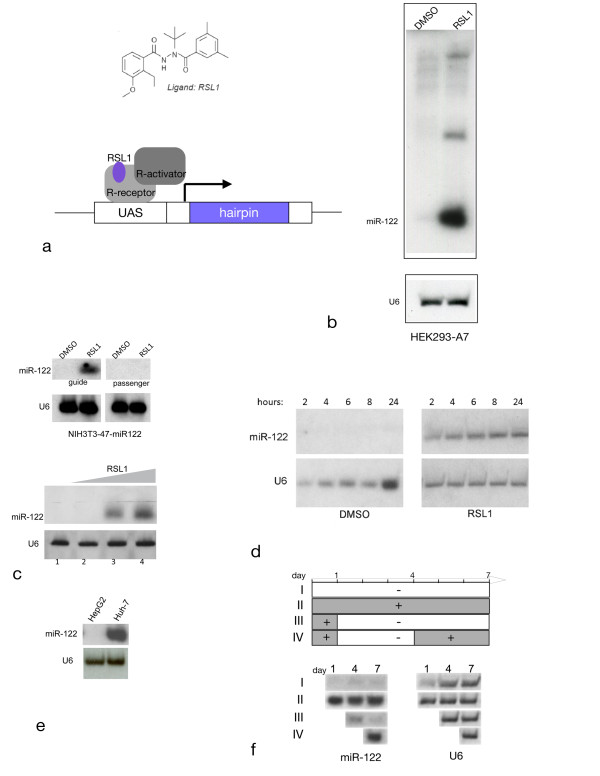
**Characteristics of inducible miR-122 expression with Rheoswitch**. a. RSL1 structure and schematic representation of the Rheoswitch system. b-f: Northern blot analyses of total RNA from non-induced (DMSO) and induced (RSL1) cells treated as indicated for each panel. b. HEK293-A7 cells transiently transfected with pNEBRX-1 containing a genomic miR-122 fragment. c. Top panel: Production of miR-122 guide vs. passenger strand: stably transformed NIH3T3-47-miR122 cells were treated as indicated. Bottom panel: RSL1 concentration-dependent expression of miR-122. Cells were treated with DMSO (lane 1) or increasing concentrations of RSL1 (50 nM, 500 nM or 5 μM, lanes 2-4)Probe: miR-122 guide strand. d. Time course of induction: RNA was prepared from cells at times indicated, beginning 2 hours after addition of DMSO (left) or RSL1 (right). Note that although the 24 h non-induced cell RNA sample is overloaded no miR-122 guide strand is detected. e. miR-122 expression in human hepatocarcinoma derived HepG2 and Huh7 cell lines. f. Switch on/off properties. Top: schematic representation of treatment regimen and sampling times. Each row (I to IV) of the scheme corresponds to a row on the northern blot below. Time is indicated as days elapsed, + RSL1 (shaded) and - RSL1 (clear). Bottom: Northern blot of total RNA hybridized with miR-122 guide strand probe (left) or U6 control (right). See Methods for probe sequences.

Pol II based expression systems offer better control through the use of tissue specific or conditional promoters. A variety of regulated systems have been developed for inducible gene expression (see [13] for review) but perhaps the most widely used are the doxycycline and ecdysone controlled systems [14]. An improved version of the ecdysone-inducible approach is a two-hybrid version, also known as the Rheoswitch system, which uses an artificial heterodimeric nuclear receptor for ligand-induced transcription of a gene cloned into an expression plasmid [15]. Two modified nuclear receptors, "RheoReceptor-1" and "RheoActivator", driven by constitutive promoters are carried on one plasmid. The "RheoReceptor-1" is a fusion of the GAL4 DNA binding domain with a modified ecdysone receptor (EcR) ligand binding domain. The "RheoActivator" is a fusion of the viral transcription activation domain VP16 with a chimeric mammalian/insect RXR ligand binding domain. The transcription unit of interest is cloned downstream of five GAL4 response elements (UAS) in a separate expression plasmid [15] (Figure 1a). Instead of ecdysone, a non-steroidal diphenylhydrazine compound, RSL1, acts as a specific ligand that stabilizes the nuclear receptor heterodimer and activates transcription of the cloned gene of interest. This combination of chimeric receptors with a non-steroidal synthetic ligand was designed to ensure that the expression system will not interfere with endogenous cellular pathways [16]. RSL1 (as opposed to ecdysteroids) has shown minimal effects on endogenous gene expression and cell proliferation in prostate cells [17] and in HEK293 cells [18]. In addition to these systems, Rheoswitch has been used to induce expression of proteins in mice, MBT-2 and Panc02 carcinoma cells [19] and NIH3T3 cells [15]. However, no gene silencing studies using this system have yet been published. In this study, we used Rheoswitch to produce RNAs that are processed *in vivo *in mammalian cultured cells to generate miRNAs that are functional in target gene knockdown.

## Results and Discussion

### Inducible expression of miR-122

We chose miR-122, an abundantly expressed miRNA, as a model for inducible shRNA expression. miR-122 is expressed exclusively in the liver [20] and plays a key role in the regulation of cholesterol and fatty acid metabolism in the adult liver [21]. In hepatocarcinoma of humans and rodents, miR-122 has been reported to be specifically down-regulated to a significant degree [22]. Hepatoma cell lines expressing miR-122, such as Huh-7, are required for the propagation and study of hepatitis C virus (HCV). HepG2, which does not express detectable miR-122 is resistant to HCV infection [23]. Recently, the first miRNA-based drug has been shown to be protective against HCV infection in primates [24]. These properties of miR-122 have made it a highly studied miRNA and a prime target for therapeutics development.

To determine whether the Rheoswitch expression system could be used to induce expression of short hairpin RNAs, a 385 bp human genomic DNA fragment containing miR-122 and its flanking DNA sequence was cloned downstream of the GAL4 binding sites in the Rheoswitch expression vector pNEBR-X1. Transient transfection of this plasmid into human embryonic kidney cells stably expressing RheoReceptor and RheoActivator (HEK293-A7) demonstrated production of mature miR-122 upon induction with RSL1 (Figure 1b). Based on these results and additional transient transfection experiments (data not shown), we constructed a cell line that carried integrated miR-122 expression vector (NIH3T3-47/miR-122) in mouse embryo fibroblasts, which also stably express the Rheoswitch proteins. We used this cell line to investigate the properties of induced miR-122 expression.

First, we confirmed that RSL1 treatment could induce the cells to produce mature miR-122 miRNA. Northern blot analysis demonstrated that RSL1-treated cells produced the 23 nt guide strand, whereas the passenger strand was undetectable, indicating that the miR-122 primary transcript is induced and correctly processed (Figure 1c, top panel). In the absence of RSL1, cells did not produce any detectable passenger or guide strand. Induction of the cells for 24 hours using different concentrations of RSL1 showed that the amount of accumulating mature miR-122 could be modulated by the concentration of the inducer (Figure 1c, bottom panel). One advantage of small molecule ligands such as RSL1 is the ability to diffuse into cells to rapidly induce, or turn off expression, in a dose-dependent manner [19]. To study these features for miRNA expression, an examination of the induction time course and ON-OFF switching was performed. miR-122 guide strand was detectable 2 hours after induction, its level peaked at about 8 hours post-induction and remained at that level after 24 hours, indicating rapid induction and steady levels of miRNA production (Figure 1d). These results demonstrate that, in principle, the NIH3T3-47/miR-122 cell line can become miR-122 positive or negative, depending on the presence or absence of RSL1 in the culture medium, mimicking Huh-7 or HepG2 hepatic cells respectively in terms of miR-122 expression status (Figure 1e).

We studied long-term expression and switching properties by maintaining the cells under non-induced or continuous induction conditions for several days. Non-induced cells showed no detectable miR-122 accumulation after 7 days in culture, while induced cells expressed miR-122 for at least 7 days, demonstrating tight control of the OFF state (Figure 1f, rows I and II). Next, we tested the reversibility of the switch. Cells were treated with RSL1 for 24 hours, RSL1 was then withdrawn. miR-122 expression was reduced over the course of the next days, reaching non-induced levels by day 7 (Figure 1f, row III). A second induction at day 4 can restore expression to the fully induced levels (Figure 1f, row IV). These results demonstrate that induced expression of miRNA using this system is sustainable and reversible, allowing control of expression of the cloned miRNA.

#### Inducible and regulated silencing

**Figure 2:**
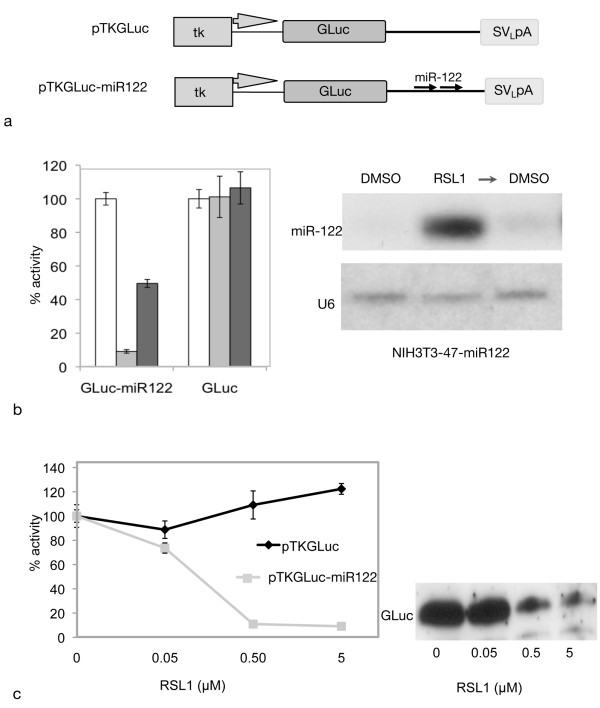
**GLuc-miR-122 reporter knockdown by inducible short hairpin expression is reversible and RSL1 concentration-dependent**. a. *Gaussia *luciferase (GLuc) reporters used in transfections: pTKGLuc: *Gaussia *luciferase under control of the constitutive HSV-TK promoter; pTKGLuc-miR122 carries two tandem miR-122 targets (arrows) inserted in the 3' untranslated region of GLuc. b. Left panel: NIH3T3-47/X1-miR122 cells were transfected with pTKGLuc-miR122 or pTKGLuc and treated with DMSO (white bars) or 0.5 μM RSL1 (gray bars) for 48 h, or 0.5 μM RSL1 for 24 h followed by DMSO (RSL1/DMSO, black bars) for 24 h. GLuc reporter activity was assayed from transfected cell culture supernatants and RNA was prepared from the same cells. Values are expressed as a percent of the mean GLuc activity of non-induced cells (+/- 1SD). Right panel: Northern blot analysis of RNA prepared from the cells transfected and treated as shown in Left panel. Probes: miR-122 guide strand (top), U6 loading control (bottom). c. NIH3T3-47/X1-miR122 cells were transfected with pTKGLuc-miR122 or control pTKGLuc plasmid, and treated with DMSO or increasing concentrations of RSL1. Mean GLuc activity of induced cells is expressed relative to mean GLuc activity of non-induced (DMSO-treated) cells (+/- 1SD). Western blot: cell culture supernatants from c, detected with anti-GLuc antibody. Lane 1: DMSO; lanes 2-4: 0.05 μM, 0.5 μM and 5.0 μM RSL1 respectively.

Since this system proved suitable for controlled expression of miR-122, we tested whether the induced miRNA expression can be used in turn for controlled target gene silencing. First, we designed a reporter-based assay using the secreted *Gaussia *luciferase (GLuc). We had previously used a GLuc-based reporter for assessing siRNA potency [25]. Two tandem copies of a sequence complementary to the miR-122 guide strand were inserted into the 3' UTR of a GLuc reporter (pTK-GLuc-miR122) (Figure 2a). The miR-122 guide strand should work, in this instance, like an siRNA because it perfectly matches its target in the 3' UTR of the GLuc mRNA. We transfected pTK-GLuc-miR122 or control pTK-GLuc reporter into the NIH3T3-47/miR-122 cells and measured the secreted luciferase activity after different treatments. GLuc expression was unaffected in non-induced cells, but was substantially reduced following induction of miR-122 expression (Figure 2b, left panel). Consistent with the reversible miR-122 expression (shown above in Figure 1f), GLuc expression was restored following withdrawal of RSL1 (Figure 2b, left panel), demonstrating reversible knock down of the target. Knockdown of GLuc expression was target-specific since the control reporter lacking the miR-122 target sites was unaffected by any treatment (Figure 2b, left panel). Since the secreted luciferase assay is non-destructive, we used the same cells to correlate the expression status of the miR-122 guide strand detected by Northern hybridization. Consistent with the luciferase expression level, miR-122 was detected only under inducing conditions (RSL1 treatment). Following RSL1 withdrawal, miR-122 declined over the next 24 hours (Figure 2b, right panel).

Since this system showed regulation of miRNA output by varying the concentration of the inducer (see Figure 1c), we tested whether the downstream effects of miR-122 expression were also RSL1 concentration-dependent. When expression of the pTK-GLuc-miR122 reporter was assayed after treatment with increasing concentrations of RSL1, a corresponding decrease in *Gaussia *luciferase activity was observed, whereas the activity from cells transfected with the control vector produced normal amounts of luciferase (Figure 2c). Western blot analysis confirmed that the loss of measured luciferase activity reflects the decreased GLuc protein levels as a result of miRNA targeting (Figure 2c). These results demonstrate that miR-122 short hairpin-mediated target silencing can be controlled in an RSL1 dose-dependent manner.

#### Induced silencing of miR-122 target genes

**Figure 3:**
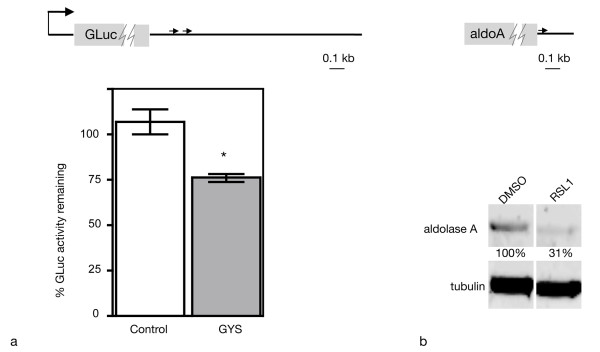
**Knock down of target genes by induced expression of miR-122**. a. Glycogen synthase-3' UTR targeted by miR-122. NIH3T3-47/X1-miR122 cells were transfected with reporter plasmids pTKGLuc (control), or pTKGLuc-GYS with the (glycogen synthase 3' untranslated region (UTR) and mIR-122 target sites schematic, top panel). The luciferase activity remaining 48 h after induction is plotted as a percent of activity from control cells. (*p = 0.0255; Error bar = -/+1SD) (See Methods for 3' UTR sequence coordinates.) b. Western blot analysis of aldolase A, an endogenous target of miR-122,. NIH3T3-47/X1-miR122 cells were treated with DMSO or 0.5 μM RSL1, then cell lysates were used for western blot analysis. The sample of 9 days post treatment is shown. Aldolase A protein quantification was calculated after LiCor scanning of Western blot normalized for loading with alpha-beta tubulin. A single miR-122 target site in aldo A is located at position 27-34 in the aldoA 3' UTR (top panel).

We demonstrated that induced expression of the guide strand of miR-122, acting as an siRNA, can silence an artificial reporter gene with a perfectly matched target sequence present in its 3' UTR (Figure 2b). To test whether the system could be used for "natural" miRNA target validation we attempted to recapitulate the silencing activity of miR-122 through its interaction with the 3'-UTR of previously identified target genes such as glycogen synthase (GYS) [21]. The 3' UTR of GYS was cloned downstream of GLuc in the pTK-GLuc reporter vector and the resulting construct (pTK-GLuc-GYS) was transfected into the miR-122 expressing stable cell line NIH3T3-47/miR-122. Upon RSL1 induction, *Gaussia *luciferase activity from cells transfected with the GYS reporter was reduced to 76% of control expression (Figure 3a). This knockdown is target sequence-specific since luciferase activity from cells transfected with pTK-GLuc control plasmid with an unrelated UTR was unaffected by miR-122 induction (Figure 3a). A similar reduction was observed using GLuc reporter assays for another miR-122 target, CAT1 [26] (data not shown).

In order to further test this miRNA target validation methodology with an endogenous (not transfected) gene target, we tested the effect of induced miR-122 expression on aldolase A, a validated miR-122 target in mouse liver, [21]. We confirmed by Western blot analysis that aldolase A is expressed in uninduced NIH3T3-47/miR-122 cells (Figure 3b, DMSO lane). We monitored aldolase-A protein levels by immunoblot over the course of miR-122 induction. In the presence of RSL1 NIH3T3-47/miR-122 cells show a gradual reduction of aldolase A protein to 75% of control in 3 days and 31% of control levels after 9 days compared to time-matched non-induced NIH3T3-47/miR-122 control cells (Figure 3b, RSL1 lanes, and data not shown). Thus we achieved modulation of aldolase-A protein expression with small molecule induction of ectopic miR-122 expression. These results suggest that miRNA target gene validation and phenotypic analysis can be easily obtained using this inducible miRNA system.

#### Expression of inducible shRNA

**Figure 4:**
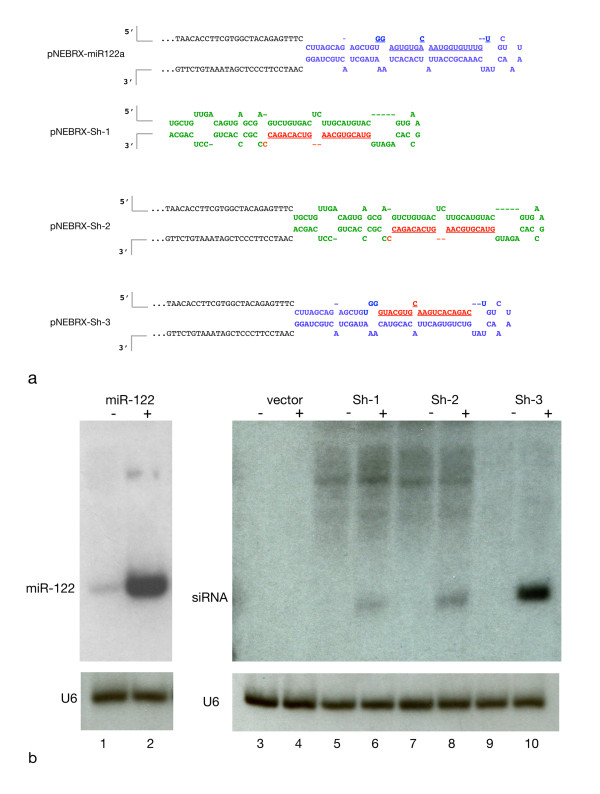
**Test of different hairpin designs reveals most efficient processing of small RNA from short hairpins based on the miR-122 structure**. a. Sequence of different short hairpin RNAs expressed from the inducible pNEBR-X1 vector. miR122 stem-loop (blue); surrounding genomic DNA sequence (black); MCS of the vector (gray lines); a short hairpin based on miR-30 (green), carrying a new guide strand sequence (red). b. Northern blots of resulting short RNAs from the short hairpin plasmids described in (a) or empty vector. The plasmids were transfected into NIH3T3-47 cells, which were treated with 0.5 μM RSL1 or DMSO for 48 h. Short hairpins were detected with probes complementary to the guide strand for Sh-1, -2, -3 or miR-122, respectively; U6 hybridization was used as loading control. (See Methods for probe sequences.)

To test whether artificial short hairpins could be expressed and properly processed to produce a designed guide strand, different short hairpin configurations carrying the same inserted guide strand sequence were cloned in the Rheoswitch expression vector pNEBRX1 (Figure 4a). The sequence and structure of the short hairpins in pNEBRX-Sh-1 and pNEBRX-Sh-2 were modeled on miR-30 as previously described [8]. In pNEBRX-Sh-1, the short hairpin sequence is cloned directly into the MCS of the vector. The hairpin sequence in pNEBRX-Sh-2 is the same as pNEBRX-Sh-1, but it is inserted in the place of miR-122 in the 385 bp genomic DNA fragment used above for miR122 expression. pNEBRX-Sh-3 uses the structure of the miR-122 short hairpin but it contains the same guide strand as Sh-1 and Sh-2. Compensatory changes were made in the stem sequence in order to maintain a miR-122-like (bulged) structure. Plasmids encoding these short hairpins were transfected into NIH3T3-47 cells, expression was induced by RSL1 and Northern blot analysis using guide strand-specific probes was used to evaluate hairpin processing. Sh-1 produced little RNA of the expected size(21-23 nt), perhaps because the stem-loop structure was not conducive to optimal processing (Figure 4b). Sh-2 produced more, suggesting that processing is more efficient if the short hairpin is surrounded by miR-122 genomic sequences. Sh-3, which most closely mimics miR-122 produced the most mature guide strand (Figure 4b). These results suggest that the miR-122 stem loop structure more readily accommodates guide strand sequence variants than the miR-30 stem loop structure.

#### Inducible siRNA production for controlled target knock down

**Figure 5:**
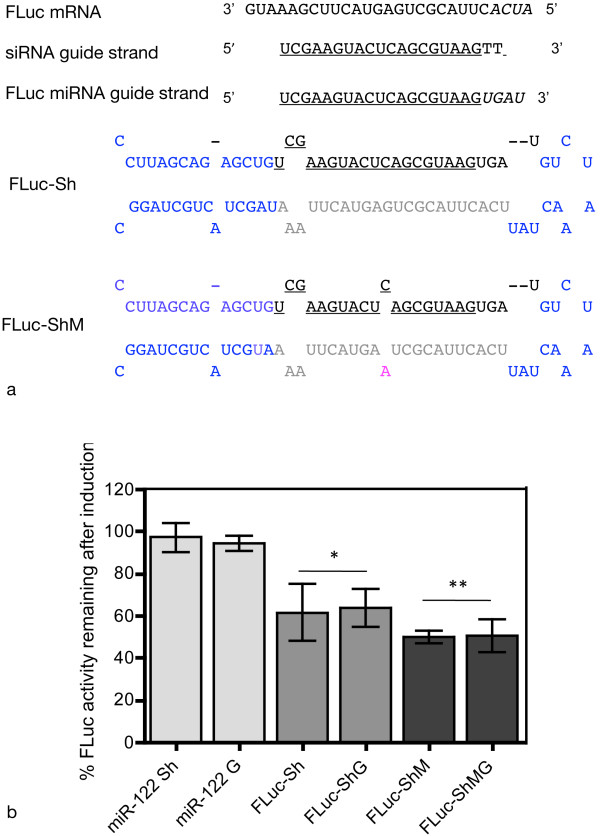
**Short hairpins based on miR-122 designed to express a particular siRNA guide strand**. An siRNA sequence used previously to silence firefly luciferase (FLuc) was placed in the miR-122 stem-loop structure as shown schematically. a. Top: The sequence of the FLuc target mRNA (shown 3' to 5') was used to extend the complementary siRNA guide strand (middle underlined). Bottom: Schematic representation of the two short hairpins designed to express firefly luciferase siRNA. The 19 nt sequence of the FLuc siRNA guide strand is underlined. The sequence of the miRNA expected from these short hairpins is 23 nt in length (black). FLuc-ShM contains a mismatch (magenta). Sequence residues originating from the miR-122 pre-miRNA are blue. All hairpin forms are designed to produce identical guide strand sequences. b. Inducible silencing of firefly luciferase. FLuc short hairpin constructs were transiently transfected into NIH3T3-47 cells along with the Fluc reporter plasmid pGL3Luc and pCMV-lacZ as a transfection control. Cells were treated with 0.5 μM RSL1 or DMSO for 48 h, and cell lysates were subsequently assayed for firefly luciferase and β-galactosidase for normalization. The luciferase activity remaining 48 h after induction is shown as a percent of activity from matched transfected non-induced cells. Results represent the mean (+/- SD) of 3 experiments. p-values represent comparison of percent remaining activity for FLuc short hairpins vs. non-targeting control miR-122 short hairpins. * p < 0.01, ** p < 0.001.

One hurdle in using an inducible shRNA expression systems is to reliably convert a desired siRNA into an inducible hairpin that can silence target genes. This process is not always straightforward. We tested whether the miR-122 backbone in the Rheoswitch expression vector could be used as a platform for inducible expression of a shRNA with a desired sequence for RNAi silencing. To test the system, we chose a previously described siRNA directed against a 19 nt sequence of firefly luciferase (pGL3-FLuc) [27]. Based on our previous results (Figure 4), we replaced the 23 nt guide strand of miR-122 in pNEBRX-miR-122 with the FLuc siRNA guide strand in a structure similar to Sh-3 (Figure 4a). Because the published (matching) guide strand sequence was 19 nt long, four nucleotides shorter than the miR-122 guide strand, we added four nucleotides (derived from the sequence of firefly luciferase) to the 3' end of the FLuc guide strand in order to maintain the structure of the miR-122 short hairpin (Figure 5a). The miR-122 stem contains a bulge, so we designed one short hairpin, FLuc-ShM, with an internal mismatch in order to mimic the miR-122 structure. A second FLuc short hairpin, FLuc-Sh was designed with a perfectly complementary stem structure (Figure 5a). Additionally, based on our Northern blot results with Sh-2 (Figure 4a, b), we tested whether surrounding the FLuc short hairpin with miR-122 genomic flanking sequence has an effect on its silencing properties. Therefore, we inserted both the mismatched and perfectly complementary FLuc short hairpins into the Rheoswitch expression vector either surrounded by genomic sequences (FLuc-ShMG and FLuc-ShG) or directly cloned into the MCS of the vector (FLuc-ShM, FLuc-Sh).

The FLuc short hairpin plasmids or an equivalent miR-122 short hairpin plasmid (miR122Sh or miR122G) were co-transfected with an FLuc reporter plasmid into Rheoswitch cells and compared for induced knockdown of firefly luciferase. All of the FLuc short hairpin designs were effective in reducing luciferase activity upon RSL1 induction (Figure 5b) while the control miR-122 had no effect on the luciferase reporter activity. The stem-mismatched short hairpins (FLuc-ShM, FLuc-ShMG), which more closely mimic the miR-122 structure, were slightly more effective in knocking down the FLuc reporter than the perfectly matched hairpin designs (FLuc-Sh, FLuc-ShG). The surrounding DNA context had no significant effect on the knockdown obtained by either FLuc short hairpin, i.e., short hairpins flanked by genomic sequences were neither more nor less effective than those flanked by the MCS of the vector (Figure 5b).

Taken together these results provide guidance in designing shRNA expression constructs. Short hairpins based on the miR-122 stem loop structure can produce more miRNA guide strand than short hairpins based on the miR-30 structure (Figure 4b, compare miR-122 and Sh-3 to Sh-1 and Sh-2). If the short hairpin was not readily processed, as was the case with the mir-30-like Sh-1, addition of flanking sequence increased the processing efficiency (compare Sh-2 to Sh-1). Neither Sh-1 nor Sh-2 produced as much miRNA as the miR-122 short hairpin or the miR-122-like Sh-3. It seems probable that Sh-3 produced more mature miRNA than Sh-1 and Sh-2 because it is a variant of the miR-122 short hairpin, rather than because of the genomic DNA that flanks it (Figure 4a).

The designs with the bulge in the stem (FLuc-ShM and FLuc-ShMG), mimicking the structure of miR-122, were slightly more effective in target knockdown than those perfectly complementary (FLuc-Sh and FLuc-ShG). The target knockdown results support the conclusion that in determining the processing efficiency and silencing effectiveness in the structure of the short hairpin is more important than the sequence context in which it is transcribed, and the miR-122 stem-loop structure is a favorable vehicle for short hairpin expression.

## Conclusions

We have shown that in addition to controlled protein expression, the Rheoswitch ligand-inducible system allows regulated expression of short hairpin RNA. By varying the dose of RSL1, the RNA expression level can be modulated, and upon RSL1 withdrawal, expression is turned off. This feature is important when studying biological phenomena resulting from down-regulation, but not elimination, of gene function. Expression can be turned on and off repeatedly, allowing additional control for studying a range of experimental states, an advantage when studying essential genes. Short hairpins can be expressed and the RNA processed to yield miRNA (miR-122) or siRNA (FLuc). The expressed miRNAs function as expected in target knockdown using endogenous targets, such as aldolase-A, or reporter-3' UTR targets (e.g., GYS), facilitating miRNA target validation assays. We explored whether novel guide strand sequences, such as those based on an siRNA, can be incorporated into the miR-122 short hairpin, expressed and processed to yield functional small RNAs. Our experiments suggest that the miR-122 backbone can be adapted for inducible siRNA expression. It has been shown that multiple shRNAs, directed at multiple targets, can be expressed from a single transcription unit [28]. The Rheoswitch system accommodates long transcription units, unlike pol III systems that require short transcripts. This suggests that it may be possible to build a Rheoswitch expression vector with two or more shRNAs in tandem.

## Methods

### Construction of short hairpins in Rheoswitch expression vector pNEBRX1

pNEBRX1-miR-122: A 385 bp miR-122 genomic fragment was generated by PCR using 2× Taq mix (NEB) and human genomic DNA (Novagen) using the following primers:

5' GTCACTAAGCTTCAGCTCTTCCCATTGCTCAAGATGC 3' and

5' GTCACTGGATCCGTGAGAGGCAGGGTTCAGCTAACCA 3'.

Vector pNEBR-X1(puro) was obtained by cloning a puromycin resistance cassette (PvuII-BamHI fragment) from pPur (BD Biosciences) into pNEBR-X1 (NEB). The miR-122 genomic PCR product and vector pNEBR-X1(puro) were digested with HindIII and BamHI and ligated together.

pNEBRX1-FLuc-Sh, pNEBRX1-ShM, pNEBRX1-miR-122sh and pNEBRX-Sh-1 were constructed by first annealing complementary oligonucleotides. Top and bottom oligonucleotides (50 pmoles each) were annealed by heating to 95°C and cooling slowly to 25°C in 10 mM TRIS pH 8.0. The resulting double-stranded DNA fragments with cohesive ends were ligated to appropriately digested pNEBR-X1, and transformed into competent *E. coli *strain NEB10beta (NEB). The following oligonucleotides were used:

FLuc-Sh BamHI top:

GATCCCCTTAGCAGAGCTGTCGAAGTACTCAGCGTAAGTGATGTCTAAACTATT

CACTTACGCTGAGTACTTAAATAGCTACTGCTAGGCC

FLuc-Sh XhoI bottom: TCGAGGCCTAGCAGTAGCTATTTAAGTACTCAGCGTAAGTGAATAGTTTAGA

CATCACTTACGCTGAGTACTTCGACAGCTCTGCTAAGGG

FLuc-ShM BamHI top: GATCCCCTTAGCAGAGCTGTCGAAGTACTCAGCGTAAGTGATGTCTAAACTATTCACTTACGCTAAGTACTTAAATAGCTACTGCTAGGCC

FLuc-ShM XhoI bottom:

TCGAGGCCTAGCAGTAGCTATTTAAGTACTTAGCGTAAGTGAATAGTTTAGACATCACTTACGCTGAGTACTTCGACAGCTCTGCTAAGGG

miR-122Sh BamHI top: GATCCCCTTAGCAGAGCTGTGGAGTGTGACAATGGTGTTTGTGTCTAAACTATCAAACGCCATTATCACACTAAATAGCTACTGCTAGGCC

miR-122Sh XhoI bottom:

TCGAGGCCTAGCAGTAGCTATTTAGTGTGATAATGGCGTTTGATAGTTTAGACACAAACACCATTGTCACACTCCACAGCTCTGCTAAGGG

pNEBRX-Sh-1 HindIII top:

GCTAAAGCTTTGCTGTTGACAGTGAGCGAGTCTGTGACTCTTGCATGTACGTGAAGCCACAGATG

pNEBRX-Sh-1 BamHI bottom:

TAGCGGATCCTGCTGAGGCAGTGGGCGGGGTCTGTGACTTGCACGTACCATCTGTGGCTTCAC

### USER cloning of- pNEBRX-Sh-2, pNEBRX-Sh-3, FLuc-ShG, FLucShMG

The precise substitution of, Sh-2 Sh-3 and FLuc short hairpins for the miR-122 short hairpin, without changing the surrounding genomic DNA was accomplished using USER technology (NEB) [29]. The plasmid vector is derived from pNEBRX1-miR-122 and contains all the sequences of the original plasmid except the miR-122 short hairpin and was generated by whole plasmid inverse PCR with the following primers containing USER sites (underlined):

5' AAACTCTG**U**AGCCACGAAGGTGTTAACTTCACCT 3' and

5' AATCC**U**TCCCUCGATAAATGTCTTGGCATCGTTTGC 3'.

The short hairpin inserts were constructed by annealing and extending oligonucleotides (listed below) with USER sites corresponding to the vector at their 5' ends (underlined), and short regions of complementarity at their 3' ends, .50 pmoles of top oligo was annealed with 50 pmoles of bottom oligo in 10 mM Tris pH7.2, by heating to 95°C for 5 minutes, then cooling slowly to 25°C. Oligonucleotides were extended using Pfu Turbo Pol Cx (Stratagene).

Vector and insert were mixed, digested with USER enzyme for 15 minutes at 37°C, annealed for 15 minutes at 25°C, then transformed into competent *E. coli *strain NEB5alpha. (NEB).

Oligonucleotide sequences for USER cloning:

Sh-2 top:

ACAGAGTT**U**TGCTGTTGACAGTGAGCGAGTCTGTGACTCTTGCATGTACGTGAAGCCACAGATG

Sh-2 bottom:

AGGGAAGGAT**U**TGCTGAGGCAGTGGGCGGGTCTGTGACTTGCACGTACCATCTGTGGCTTCAC

Sh-3 top:

ACAGAGTT**U**CCTTAGCAGAGCTGTGGGTACGTGCAAGTCACAGACTGTCTAAACTATGTC

Sh-3 bottom:

AGGGAAGGAT**U**GCCTAGCAGTAGCTATTTGTACGTGTAAGTCACAGACATAGTTTAGACAGT

Fluc-ShG top: ACAGAGTT**U**CCTTAGCAGAGCTGTCGAAGTACTCAGCGTAAGTGATGTCTAAACTAT

FLuc-ShG bottom: AGGGAAGGAT**U**GCCTAGCAGTAGCTATTTAAGTACTCAGCGTAAGTGAATAGTTTAGAC

Fluc-ShMG top: ACAGAGTT**U**CCTTAGCAGAGCTGTCGAAGTACTCAGCGTAAGTGATGTCTAAACTAT

Fluc-ShMG bottom: AGGGAAGGAT**U**GCCTAGCAGTAGCTATTTAAGTACTTAGCGTAAGT GAATAGTTTAGAC

### Cloning glycogen synthase (GYS) 3' UTR

GYS 3'UTR was cloned by from HEK293-A7 cell polyA+ RNA by RT-PCR using the Protoscript First Strand cDNA Synthesis kit (NEB) and 2× Taq mix (NEB). The GYS PCR primers contained USER enzyme (NEB) cleavage sites. RT-PCR products were cleaved with USER enzyme (NEB), mixed with pNEB206A USER vector and transformed into competent *E. coli *strain NEB5alpha (NEB). The resulting plasmid was digested with NotI and XhoI and the 3'UTR-containing fragment was ligated into the NotI and XhoI sites of pTK-GLuc. The following oligonucleotide primers were used: GGGAAG**U**GCGGCCGCGTCCGCCCCACCACACTCCCCGCCTGTC (2395-2422) and GGAGACA**U**ACCGGTTCATCTCATCTCCGGACACACTCCATTCA (3528-3500). Coordinates are from human GYS sequence, accession number NM_002103.

### Cloning miR122 target into reporter plasmid pTK-GLuc

Oligonucleotides encoding two direct repeats of a sequence complementary to the miR-122 guide strand were annealed by heating to 95°C and cooling slowly to 25°C in 10 mM TRIS pH7.2. The resulting double stranded DNA fragment containing NotI and XhoI cohesive ends was ligated to pTK-GLuc (NEB) digested with NotI and XhoI to produce pTK-GLuc-miR122. Oligonucleotide sequences:

GCGGCCGCACAAACACCATTGTCACACTCCAAATCACACAAACACCATTGTCACACTCCAC and TCGAGTGGAGTGTGACAATGGTGTTTGTGTGATTTGGAGTGTGACAATGGTGTTTGTGC

All restriction endonucleases were obtained from New England BioLabs (NEB).

### Cell culture

NIH3T3-47, HEK-293-A7 Rheoswitch cells (NEB), and NIH3T3-47/miR122 cells were cultured in DMEM (HyClone) supplemented with 10% fetal bovine serum (FBS), 1× non-essential amino acids, 2 mM L-glutamine, and 800 μg/mL geneticin (G418) (all from GIBCO). In addition, NIH3T3-47/miR122 cells were cultured with 1 μg/ml puromycin (Sigma). Cells were grown at 37°C, in 5% CO_2 _atmosphere.

### NIH3T3-47/X1-miR122 (puro) stable cell lines

NIH3T3-47 Rheoswitch cells were plated in DMEM with 10% FBS (as described) in 100 mm plates. Cells were transfected at approximately 50% confluence with 15 μg pNEBRX1-miR122 (puro) per plate. 24 hours post-transfection, cells were treated with 1 μg/mL puromycin (Sigma). Cell culture medium was changed as necessary until colonies formed. Colonies were expanded and tested for RSL1-inducible expression of miR-122 by northern blot hybridization. The stable cell lines were cultured as described above with the addition of 1 μg/mL puromycin (Sigma).

### Transfection and induction

For miR-122 target knockdown experiments, NIH3T3-47/miR122 cells were plated as described in 12 well plates and transfected at 50-70% confluence with 800 ng/well reporter plasmid and 100 ng/well pCMV-lacZ as a control for transfection efficiency, using Transpass D2 reagent (NEB) according to manufacturer's instructions. Reporters used were pTK-GLuc, pTK-GLuc-miR122, pTK-GLuc-GYS.

For FLuc knockdown experiments, NIH3T3-47 Rheoswitch cells (NEB) were plated as above in 24 well plates and transfected with 200 ng/well pGL3-FLuc, 100 ng/well pCMV-lacZ as transfection efficiency control and 100 ng/well of the plasmids encoding the firefly luciferase short hairpins (pNEBRX1-FLuc-Sh and -Fluc-Sh-M and pNEBRX1-FLuc-ShG and FLuc-Sh-MG).

Short hairpin expression was induced by addition of RSL1 (Intrexon) RSL1 is [(N-(2-ethyl-3-methoxybenzolyl)-N'-(3,5-dimethylbenzoytert-butylhydrazine] and has been also known as GS-E or RG-102240 [16] or GenoStat (Millipore). A 5 mM stock solution in DMSO was diluted to a final concentration of 500 nM in the culture medium, unless otherwise noted. Controls received an equivalent volume of DMSO. DMSO final concentration was 0.1% or less. Cells were induced at 3-16 hours post-transfection and cell culture supernatants were collected for assays at 48 hours post transfection unless otherwise indicated.

Repeated Induction protocol (Figure 1f). NIH3T3-47/miR-122 cells were plated in 12 well plates in complete medium supplemented with 500 nM RSL1 dissolved in DMSO, or an equivalent volume of DMSO (control medium). RNA was prepared from RSL1 and DMSO treated cells after 1, 4 and 7 days in culture. For ligand withdrawal treatment, cells were cultured in complete medium containing 500 nM RSL1 for 1 day, after which it was replaced with control medium. RNA was prepared from these cells on days 4 and 7. For re-induction treatment, cells that had undergone the withdrawal treatment were cultured in control medium until day 4, after which it was replaced with medium containing 500 nM RSL1. RNA was prepared from these cells on day 7.

Total RNA was prepared from cells using TRIZOL reagent (Invitrogen) according to manufacturer's instructions.

PAGE: 10-30 μg total RNA or 60 ng microRNA marker (NEB) in 4 M urea loading buffer was heated to 95°C for 5 minutes, then loaded on 12% polyacrylamide gels (SequaGel; National Diagnostics) pre-run in 1× TBE at 250 V for 1 hour prior to loading. Gels were stained with SYBRGold (Invitrogen) to visualize RNA, electroblotted to GeneScreen Plus (Perkin-Elmer Life Sciences) at 300 mA for 30 minutes and UV crosslinked on optimum setting (Spectorlinker XL1000, Spectronics).

### Probe synthesis

Oligonucleotide probes were labeled as follows: 0.5 pmol of probe oligo and 12.5 pmol of template oligo were mixed and heated to 95°C for 1 minute, incubated at room temperature for 2 minutes than placed on ice. 1 μl 10× NEBuffer 2, 5 U Klenow (exo-) (NEB), 3 μl alpha-^32^P-dATP (6000 Ci/mmole)(DuPont/NEN) and dH_2_O to 10 μl were added and incubated at 25°C for 1.5 h. Labeled oligo probe was purified over G-25 spin column (GE Healthcare) and heated to 95°C for 5 minutes before adding to hybridization. The following oligonucleotides were used:

Probe complementary to miR-122 guide strand: ACAAACACCATTGTCACACTCCA

miR-122 guide strand template: TTTTTTTTTTTGGAGTGTG

Probe complementary to miR-122 passenger strand: TGGAGTGTGACAATGGTGTTTGT

miR-122 passenger strand template: TTTTTTTTTTACAAACA

Probe complementary to Sh-1, Sh-2 and Sh-3 guide strand:GTCTGTGACTTGCACGTAC

Sh-1, Sh-2, Sh-3 guide strand template:TTTTTTTTTTGTACGTG

Probe complementary to FLuc guide strand: ATCACTTACGCTGAGTACTTCGA

FLuc guide strand template: TTTTTTTTTTTCGAAGT

Complementary regions of probe and template oligos are underlined.

U6 oligo probe: 5'CGTTCCAATTTTAGTATATGTGCTGCCGAAGCGA3' [30] synthesized with a biotin at each end and detected using Phototope Star Detection Kit for Nucleic Acids (NEB) according to manufacturer's instructions.

### Hybridization and detection

PAGE Northern blots were hybridized in UltraHyb Oligo (Ambion) at 37°C (U6) or 42°C (miR-122, FLuc) washed in 1% SDS, 50 mM sodium phosphate buffer pH 7.2 at 37°C or 42°C. Autoradiography was performed on Amersham Hyperfilm MP (GE Healthcare).

### Western blot

Cells were washed once in 1× PBS, followed by lysis in 1× Luciferase Cell Lysis buffer (NEB) for 15-30 minutes at 25°C with gentle agitation. Lysates were transferred to microcentrifuge tubes, cell debris was pelleted by centrifugation for 5 minutes at 4°C and lysates were stored at -20°C. 20 μL of lysate was mixed with 10 μL of 3× SDS-PAGE gel loading buffer (NEB), samples were heated to 95°C for 5 minutes and loaded on 10-20% polyacrylamide/Tris-glycine gel (Novex), run at 150 V in 1× Laemmli buffer, electroblotted to Immobilon or Immobilon-FL PVDF membrane in 1× Towbin buffer.

Tubulin control: Goat anti-alpha/beta tubulin (Cell Signaling Technologies) 1:1000 in Tris-buffered saline with 0.15% Tween20 (TBST), 2.5% milk, 2.5% BSA, followed by anti-rabbit-HRP (Cell Signaling Technologies) 1:2000. Detection used the Phototope Western detection kit (Cell Signaling Technologies);

Rabbit anti-beta tubulin (Cell Signaling Technologies) diluted 1:1000 in Odyssey buffer (LiCor) with 0.1% Tween20, followed by goat anti-rabbit-IR800 (LiCor) 1:15,000 in Odyssey buffer with 0.1%Tween 20 and 0.01% SDS. Aldolase A: Goat anti-Aldolase A (Santa Cruz Biotechnology) 1:200 in Odyssey buffer with 0.1% Tween 20 followed by donkey anti-goat-IR800, 1:15,000 in Odyssey buffer with 0.1% Tween 20 and 0.01% SDS. Fluorescent antibodies were detected using LiCor Odyssey

### Reporter Assays

All assays were done in black 96 well microtiter plates and were read using either an L-max II (Molecular Devices) or a Mithras (Berthold) luminometer. *Gaussia *luciferase assay: 20 μL cell culture supernatant was diluted with 50 μL 1× PBS, 50 μL 1× *Gaussia *Luciferase Assay reagent (NEB) was injected and a 5 second integration followed a 2 second delay. Firefly luciferase: cells were washed in 1× PBS and lysed in 1× Luciferase Cell Lysis Buffer (NEB) for 15-30 minutes at 25°C with gentle agitation. 20 μL lysate was assayed with 100 μL Luciferase Assay Reagent II (Promega Dual Luciferase Assay kit) using a 10 second integration following a 2 second delay. B-gal: cells were washed in 1× PBS and lysed in 1× Luciferase Cell Lysis Buffer (NEB) or Galacto-Light Lysis buffer (Applied Biosystems). B-gal activity was assayed using the Galacto-Light kit (Applied Biosystems) according to the manufacturer's instructions. P values were calculated using a two-tailed paired t-test.

## List of abbreviations

BP: base pair; CMV: Cytomegalovirus; ECR: ecdysone receptor; FLUC: firefly luciferase; G418: geneticin; GLUC: *Gaussia princeps *luciferase; GYS: Glycogen synthase; HCV: Hepatitis C Virus; KB: kilobasepair; LUC: luciferase; MIRNA: microRNA; MCS: multiple cloning site; NT: nucleotide residue; PBS: phosphate buffered saline; POLII: RNA polymerase II; QPCR: quantitative PCR; RSL1: Rheoswitch ligand; SHRNA: short hairpin RNA; SIRNA: short interfering RNA; SD: standard deviation; SV40: Simian virus 40; TET: tetracycline; TK: thymidine kinase; UTR: untranslated region

## Competing interests statement

The authors are employees of New England Biolabs (NEB) where this research was conducted and declare no competing interest. NEB is a manufacturer of several of the biological reagents used in this work.

## Authors' contributions

CMS carried out the experimental studies, and drafted the manuscript. GT conceived of the study, participated in its design and coordination and helped to draft the manuscript. All authors read and approved the final copy.
